# Leprosy

**Published:** 1881-03

**Authors:** 


LEPROSY.—The fact seems not to be generally known that this ancient and formidable enemy of the human races, has found its way into, and is evidently obtaining foothold in several states of this Union! The state and municipal authorities should lose no time in making efforts for the ejectment of lepers, wherever they are found within their jurisdictions. History and experience prove that where the disease once finds a lodgment, its eradication, or extermination is always attended with great difficulty. The introduction of this loathsome pestilence into the South Pacific islands, is said to have been within less than half a century, and now the early extinction of the autochthonal population is a matter of only a few generations in the future. Already the king of those islands is casting about for people to supply the places made vacant by the rapidly decimating ravages of the dreaded malady amongst his subjects. This is a note of warning which should be sufficiently audible to arouse our municipal, state, and even United States authorities, to prompt and effective efforts for its extermination from this country! The recent action of the authorities at San Francisco, in returning leprous Chinese to their native land, was highly commendable and proper, but they should exercise such vigilance as to prevent the landing of leprous subjects, if they would be kept free from the ravages of the loathsome pestilence in the future.

New York, New Orleans, and other communities in or near which the disease is said to have some subjects, should lose no time in its thorough expulsion and the extermination of any traces or germs, if possible, that may be left by its victims. This is due to the whole people of this country.

